# A 52-year-old woman with indolent verrucous plaques on a lower extremity

**DOI:** 10.1016/j.jdcr.2026.03.060

**Published:** 2026-04-08

**Authors:** Nicole A. Johnsen, Gina N. Bash, Lindsey M. Voller, Caroline Temmins, Preethi Yeturu, Harleen K. Sahni, Aruna Venkatesan, Eon J. Rios, Deeti J. Pithadia

**Affiliations:** a David Geffen School of Medicine, University of California Los Angeles, Los Angeles, California; b Department of Dermatology, Oregon Health and Science University, Portland, Oregon; c Department of Dermatology, Stanford University, Stanford, California; d Department of Anatomic Pathology & Clinical Pathology, Santa Clara Valley Medical Center, San Jose, California; e Division of Mycobacterial Diseases and International Health, Santa Clara Valley Medical Center, San Jose, California; f Division of Dermatology, Santa Clara Valley Medical Center, San Jose, California; g Division of Pediatric Dermatology, Cleveland Clinic Foundation/Cleveland Clinic Children’s, Cleveland, Ohio

**Keywords:** acid-fast bacilli, cutaneous tuberculosis, granulomatous inflammation, interferon gamma release assay, mycobacterium tuberculosis, mycobacterium tuberculosis polymerase chain reaction, paucibacillary cutaneous tuberculosis

## Case description

A 52-year-old woman presented with asymptomatic cutaneous lesions that began on the left plantar foot at age 11 and slowly ascended to the dorsal foot and lower leg. She had not sought medical care since immigrating from Mexico 20 years earlier. She denied fevers, chills, or cough. She did not recall preceding trauma but reported frequently walking barefoot outdoors.

**Fig 1:**
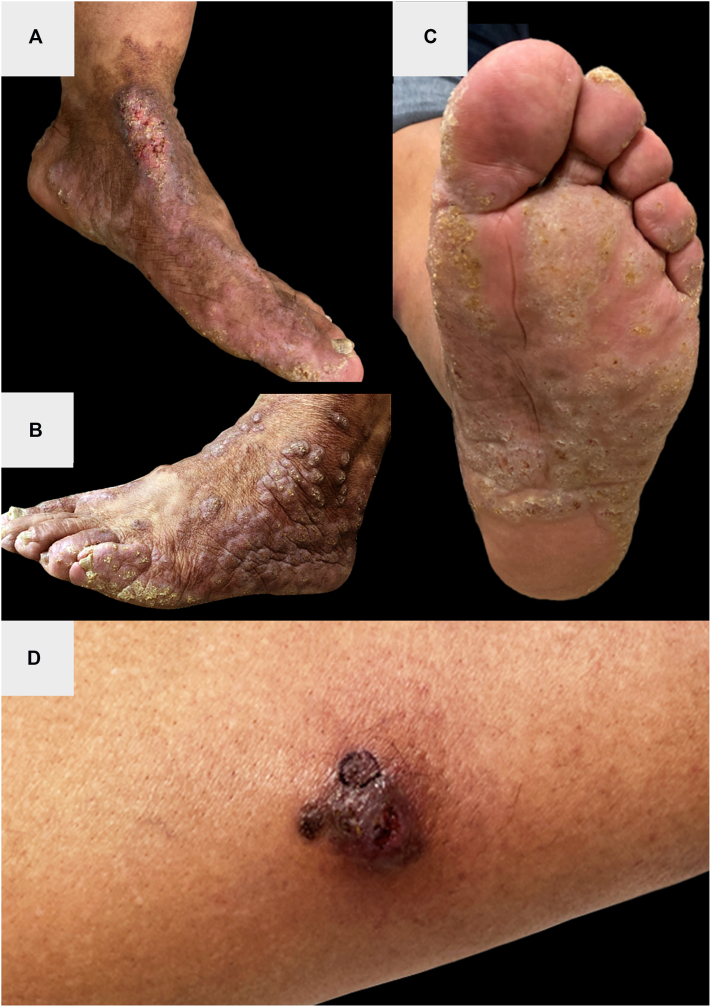
Left lower extremity at initial presentation. There were confluent erythematous-to-violaceous verrucous plaques and nodules on the dorsal foot and ankle **(A** and **B)**, yellow punctate papules coalescing into plaques on the plantar foot **(C)**, and a discrete violaceous nodule with white-silver scale on the shin **(D)**.

Examination of the left lower extremity revealed confluent erythematous-to-violaceous verrucous nodules with yellow scale on the dorsal foot and ankle (Fig 1, *A* and *B*), yellow punctate papules coalescing into plaques on the plantar foot (Fig 1, *C*), and a violaceous nodule with white-silver scale on the medial shin (Fig 1, *D*).

**Fig 2:**
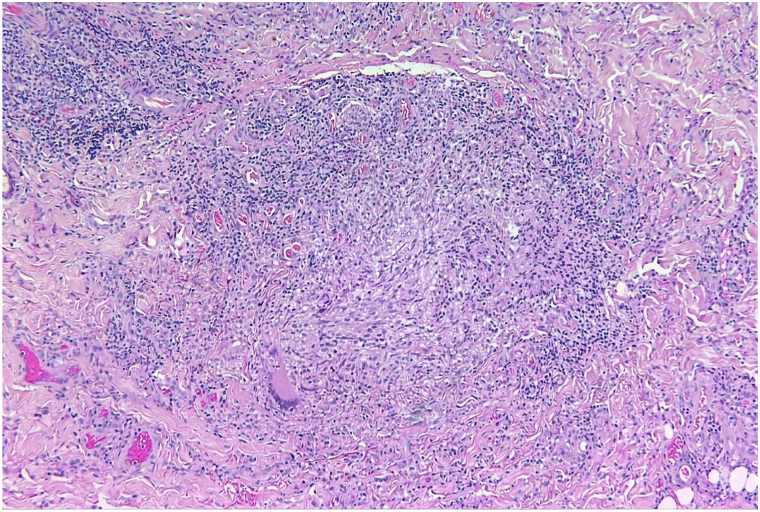
Punch biopsy taken from the left shin, demonstrating a dermal non-necrotizing granuloma comprised of histiocytes, multinucleate giant cells, and lymphocytes (hematoxylin and eosin, 100×).

Punch biopsies from the dorsal foot and shin demonstrated pan-dermal non-necrotizing granulomas composed of histiocytes, multinucleated giant cells, and lymphocytes (Fig 2). Acid-fast bacilli (AFB), Grocott methenamine silver, and Fite stains were negative. Preliminary bacterial, fungal, and AFB cultures showed no growth after 1 week.

**Question: In addition to awaiting finalized tissue culture results, what is the most appropriate next step in management?**

A. Empiric oral itraconazole therapy
B. *Mycobacterium tuberculosis* polymerase chain reaction on biopsy tissue
C. Direct immunofluorescence studies on biopsy tissue
D. Empiric oral corticosteroid therapy
E. Magnetic resonance imaging of the lower extremity

## Discussion

Correct answer: **B. *Mycobacterium tuberculosis* (MTB) polymerase chain reaction (PCR)** **on biopsy tissue**.

Given the patient’s origin from a tuberculosis-endemic region and granulomatous inflammation on histopathology, cutaneous tuberculosis (CTB) was suspected. In sensitized hosts, CTB ranges from indolent, localized verrucous plaques (tuberculosis verrucosa cutis) to progressive plaques and nodules with extracutaneous involvement (lupus vulgaris).^1^^,^^2^ These forms are often paucibacillary; bacilli may be absent on histopathology, and AFB staining and mycobacterial culture may be falsely negative.^2^, ^3^, ^4^ MTB PCR performed on biopsy tissue is the most sensitive test for CTB and can also provide information regarding drug resistance; nevertheless, false negative results remain possible in highly paucibacillary disease.^3^

If suspicion for CTB persists despite negative AFB culture and MTB PCR, repeat skin biopsies for these studies should be considered.^2^^,^^4^ Interferon-gamma release assay may also be pursued, as it can serve as a useful adjunct that may support the diagnosis of tuberculosis when tissue-directed studies are negative. If confirmatory studies remain inconclusive and clinical suspicion persists, particularly if history and histopathology are strongly suggestive, a therapeutic trial of empiric anti-mycobacterial therapy may be considered.^5^ All patients with strongly suspected or confirmed CTB should undergo screening for systemic involvement with interferon-gamma release assay, chest radiography, and other symptom-guided imaging, as extracutaneous disease may occur even in classically skin-limited variants.

**Fig 3:**
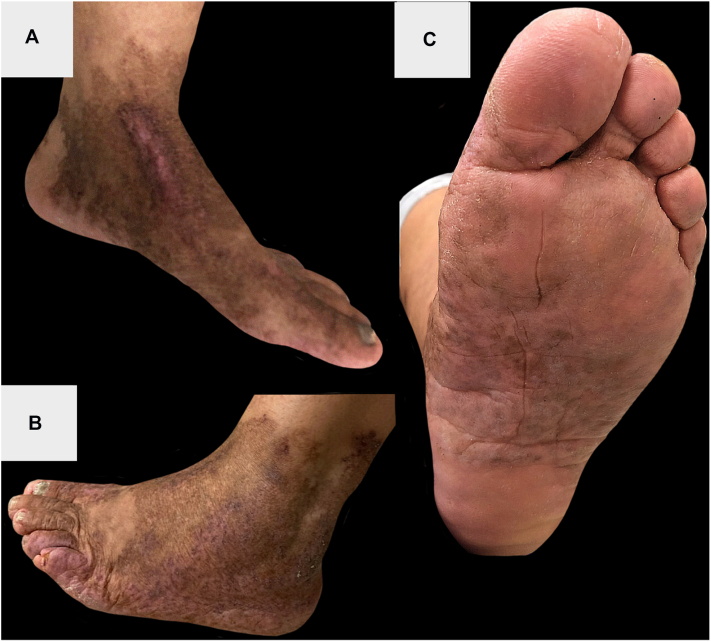
Left lower extremity following completion of anti-tuberculous therapy. The prior skin lesions resolved with hyperpigmentation, erythema, and scarring on the dorsal foot, ankle, **(A** and **B)**, and plantar foot **(C)**.

In this case, after 4 weeks of incubation, tissue AFB culture isolated MTB, confirming CTB. Interferon-gamma release assay (QuantiFERON GOLD) was positive; chest radiography was normal. She was treated with isoniazid (300 mg daily), rifampin (600 mg daily), ethambutol (1400 mg daily), pyrazinamide (1750 mg daily), and vitamin B6 (50 mg daily) for 2 months, followed by 8 months of isoniazid, rifampin, and vitamin B6 at the same dose and frequency. Following treatment completion, the lesions resolved with postinflammatory pigment changes and scarring (Fig 3).

## Conflicts of interest

None disclosed.
